# The Evolution of Dendritic Cell Immunotherapy against HIV-1 Infection: Improvements and Outlook

**DOI:** 10.1155/2020/9470102

**Published:** 2020-05-25

**Authors:** Hager Mohamed, Vandana Miller, Stephen R. Jennings, Brian Wigdahl, Fred C. Krebs

**Affiliations:** Department of Microbiology and Immunology, Center for Molecular Virology and Translational Neuroscience, Institute for Molecular Medicine and Infectious Disease, Drexel University College of Medicine, Philadelphia, PA 19102, USA

## Abstract

Dendritic cells (DC) are key phagocytic cells that play crucial roles in both the innate and adaptive immune responses against the human immunodeficiency virus type 1 (HIV-1). By processing and presenting pathogen-derived antigens, dendritic cells initiate a directed response against infected cells. They activate the adaptive immune system upon recognition of pathogen-associated molecular patterns (PAMPs) on infected cells. During the course of HIV-1 infection, a successful adaptive (cytotoxic CD8^+^ T-cell) response is necessary for preventing the progression and spread of infection in a variety of cells. Dendritic cells have thus been recognized as a valuable tool in the development of immunotherapeutic approaches and vaccines effective against HIV-1. The advancements in dendritic cell vaccines in cancers have paved the way for applications of this form of immunotherapy to HIV-1 infection. Clinical trials with patients infected with HIV-1 who are well-suppressed by antiretroviral therapy (ART) were recently performed to assess the efficacy of DC vaccines, with the goal of mounting an HIV-1 antigen-specific T-cell response, ideally to clear infection and eliminate the need for long-term ART. This review summarizes and compares methods and efficacies of a number of DC vaccine trials utilizing autologous dendritic cells loaded with HIV-1 antigens. The potential for advancement and novel strategies of improving efficacy of this type of immunotherapy is also discussed.

## 1. Introduction

Despite the demonstrated efficacy of combination antiretroviral therapy (ART), treatment of infection by the human immunodeficiency virus type 1 (HIV-1) still necessitates life-long use of ART to effectively suppress viremia in infected patients. This is partly attributed to ineffective HIV-1-specific cell-mediated immune responses due to impaired dendritic cell function in many patients on ART. Interestingly, a small percentage of infected individuals are termed “elite controllers” for their ability to control HIV-1 replication without ART. The protection from disease progression in these individuals has been attributed to robust HIV-1-specific antigen presentation and a CD8^+^ cytotoxic T-lymphocyte (CTL) response targeted against HIV-1 [1, 2]. Dendritic cell immunotherapy might have the capacity to control HIV-1 infection in the absence of ART, similar to the ability of elite controllers to do so. This type of immunotherapy involves loading dendritic cells (DCs) with antigens ex vivo then introducing the cells back into the patient. This approach has been investigated as a treatment for patients with pancreatic cancer or melanoma [3–5].

Dendritic cells have been shown to be critical to the recognition of HIV-1, regulation of T-cell function, and targeting of infected cells by activation of the adaptive immune system through presentation of HIV-1 antigens [6, 7]. The versatility of DCs in contrast with other antigen-presenting cells has been attributed to the presentation of antigens on both major histocompatibility complex (MHC) class I and MHC II molecules. Unlike other immune cells that primarily activate CD4^+^ T helper cells via MHC class II, DCs have the ability to process and cross-present HIV-1 antigens from dying cells and display them on MHC class I molecules to activate cytotoxic CD8^+^ T-lymphocytes [8–11]. In chronic HIV-1 infection, dendritic cells have been shown to be greatly reduced in number and shown to be inefficient antigen presenters [12–15]. In addition, predicting DC function is particularly difficult in the course of the disease in the elderly population [16]. While it may not be possible to enhance DC numbers, enhancement of antigen capture and presentation may be beneficial for the control of the highly variant HIV-1 population from patient to patient.

A personalized immunotherapy approach for the treatment of HIV-1 infection has thus been the aim of many recent studies, which have focused on helping the patient's own immune response better target and clear HIV-1-infected cells. To this end, clinical trials using autologous dendritic cell-based vaccines have been conducted. Similar to cancer, HIV-1 infection progresses via evasion of immune system recognition. In addition, HIV-1 in particular has been shown to compromise the immune system by exhausting T-cells. In this regard, DC immunotherapy has been focused on enhancing the induction of CTL responses [17].

The immunotherapy approach is unlike other methods of vaccination, which is aimed at eliciting broadly neutralizing antibodies usually directed against the HIV-1 structural Env protein. Accordingly, broadly neutralizing antibodies targeting regions of the HIV-1 envelope such as the V1/V2 loop, gp120 glycan residues, and the CD4 binding site have failed due to mutations that result in “escape” viruses [18–20]. A DC immunotherapy approach intended to control viral replication and disease progression, however, does not depend entirely on the neutralization of free virions. The added advantage of this approach is that it has allowed various methods of ex vivo manipulation, such as coculture systems using patient DCs with T-cells. The goal of this form of immunotherapy has been to establish a sustained T-cell response against HIV-1 in infected patients, ideally without the concern for viral rebound. In this review, the design as well as the results obtained from a number of recent clinical trials involving the use of HIV-1-specific DC vaccines will be discussed to give insights with respect to the potential of this immunotherapy approach to provide a practical tool for HIV-1 treatment.

## 2. Methods for Designing HIV-1 Antigen-Loaded Dendritic Cells *Ex Vivo*

**Figure 1:**
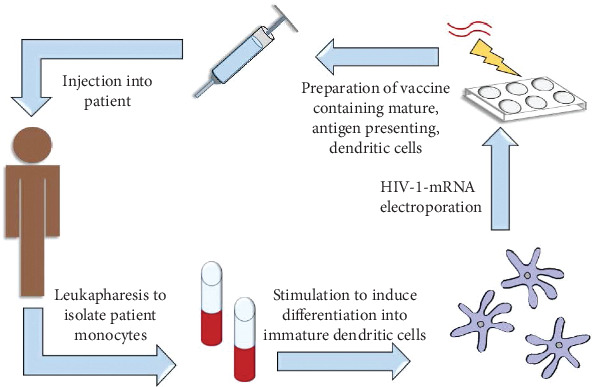
Autologous dendritic cell vaccines are prepared using the patient's own monocytes from PBMCs obtained through leukapheresis. The monocytes are stimulated *in vitro* with growth cytokines to induce differentiation into immature dendritic cells. The dendritic cells may then be loaded with HIV-1-derived antigen, commonly introduced via mRNA electroporation, after which they will become mature, antigen-presenting dendritic cells. They can then be formulated into a vaccine that is administered to the patient to elicit a T-cell response specific to the HIV-1 antigen and evoke an enhanced response against HIV-1-infected cells.

DC-based immunotherapy approaches, of course, rely on the ability to produce and manipulate dendritic cells ex vivo through the use of some well-characterized methods. Past studies have differed in the antigen choice and techniques used for delivering antigen to DCs. Studies involving DCs commonly use monocyte-derived dendritic cells obtained from HIV-1-infected patients using leukapheresis [21–23]. Monocytes are obtained from peripheral blood mononuclear cells (PBMC) and then induced to mature *in vitro* through culturing with cytokines such as granulocyte-macrophage colony-stimulating factor (GM-CSF) (Figure 1). During priming *in vivo*, GM-CSF is released by cytotoxic T-cells as a dendritic cell licensing factor, which has been shown to promote the expression of costimulatory molecules [24, 25]. Other cytokines may also be used for stimulating maturation, including interleukin-1*β* (IL-1*β*), IL-4, IL-6, tumor necrosis factor-*α* (TNF-*α*), and the interferons IFN-*α* and IFN-*γ* [12, 21–23, 26]. *Ex vivo* manipulation of DCs has the advantage of favoring a desired outcome while avoiding off target effects that may occur *in vivo*, since many cytokines function *in vivo* in feedback loops that promote opposing effects, including up- and downregulation of HIV-1 gene expression [27].

### 2.1. Choice of HIV-1 Antigens

As research has progressed into utilizing DC vaccines for HIV-1 immunotherapy, antigen loading has become an important concern. The use of HIV-1 mRNA for DC loading has become very common. Autologous antigens have in some cases been favored against general consensus sequences or synthetic antigens. Moreover, antigen combinations have become preferred by most studies over single antigens in order to address concerns of poor long-term viral suppression and lack of immunogenicity.

Dendritic cell immunotherapy approaches for HIV-1 infection have encompassed the use of both structural and nonstructural HIV-1 proteins. Structural and enzymatic proteins strictly required at specific points in the HIV-1 replication cycle are encoded by the *gag*, *pol*, and *env* genes [28, 29]. The regulatory proteins Tat and Rev, which are encoded by *rev* and *tat*, respectively [30–32], are similarly required for productive replication. The *vif*, *vpr*, *nef*, and *vpu* genes encode accessory proteins that are, in contrast, not strictly required, as shown in numerous *in vitro* systems. The inclusion of accessory proteins in vaccine design efforts partially stems from growing knowledge of their critical roles in the progression of HIV-1-associated disease despite not being strictly required during viral replication *in vitro* [33, 34].

Antigens can come from individual patients (autologous antigens) or derived synthetically from consensus HIV-1 sequences that can be found across large numbers of patients. For example, three synthetic peptides were designed based on consensus amino acid sequences at residues 386-394 in Gag, 498-506 in Pol, and 134-142 in Env [21]. Induction of T-cell responses has indeed been observed to conserved Gag sequences loaded onto dendritic cells *in vitro* in both HIV-1-infected and HIV-1-uninfected cells [35]. While the use of consensus sequences appears more convenient, it has also been associated with a lack of specific CD8^+^ T-cells elicited against patient-specific viral strains in failed clinical trials. More recent studies thus instead have investigated the use of patient-derived HIV-1 antigens and autologous Gag and accessory proteins Nef, Rev, and Vpr [35–38]. The use of HIV-1 mRNA encoding the Tat and Vpx proteins has also been studied [39, 40]. A combination of structural and nonstructural HIV-1 antigens can also be accommodated, further allowing more options for the reliance on conserved regions in each protein to target and limit variations in clinical outcomes [41]. In addition to creating a more “personalized” approach, the use of autologous antigens (Figure 1) has the benefit of allowing extraction of HIV-1 RNA from latently infected CD4^+^ T-lymphocytes that could not be cleared by ART [42].

### 2.2. Viral versus Nonviral Methods for Delivering Antigen to DCs

**Figure 2:**
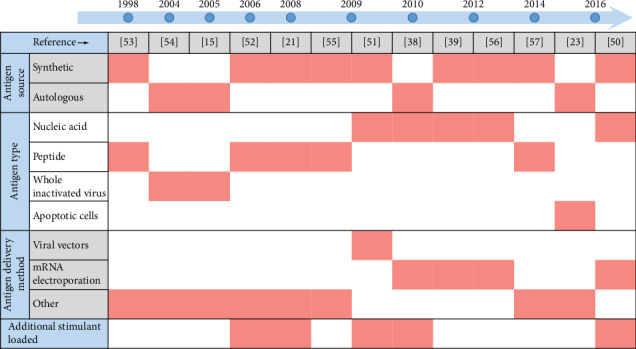
Timeline of the DC vaccine formulation design of the first clinical trials done for different studies. The DC vaccine design in these studies [15, 21, 23, 38, 39, 50–57] varied greatly in antigen type and method of delivery to DCs. More recent clinical trials predominantly investigated DC vaccines electroporated with HIV-1 mRNA. The inclusion of additional immunogens in an effort to maximize efficacy of DC function has been common.

HIV-1 RNA has been introduced into dendritic cells by nonviral and viral delivery methods utilizing different vectors. Delivery methods include the commonly used electroporation as well as viral vectors such as adenovirus and poxviruses [35, 39, 43–45]. In a recent study, dendritic cells were transduced with a lentiviral vector containing Vpx and were shown to induce multiple proinflammatory cytokines as well as an antigen-specific CTL response with donor cells *in vitro* [40]. The induction of an array of proinflammatory cytokines and chemokines was also demonstrated when DCs were transduced with the modified Vaccinia Vaccine Ankara (MVA) poxvirus vector, which has been used to simultaneously induce the maturation of monocyte-derived dendritic cells upon infection, marked by an increase in cell surface CD86 and HLA-DR expression [44]. More DC immunotherapy experiments utilizing viral vectors for *in vivo* delivery of HIV-1 RNA are necessary help to clarify their safety profiles in HIV-1 patients. Nonetheless, the HIV-1 antigen choice and loading in clinical trials over the years have shifted to HIV-1 mRNA electroporation as opposed to loading peptides, inactivated viruses, or whole cells (Figure 2). This technique, which introduces nucleic acids into the cell by transiently disrupting the cell membrane with short, high-voltage pulses, can achieve a greater than 90% transfection efficiency [46–49]. The incorporation of additional molecules used simultaneously with HIV-1 antigens in the DC vaccine design to enhance DC function has also emerged in these trials [21, 38, 50–52]. This includes the coelectroporation of DCs with RNA encoding the CD40 ligand (CD40L) to enhance the maturation and antigen presentation of DCs [38] (Figure 2).

### 2.3. Insights from DC Coculture Analysis

Following antigen introduction, manipulation of DCs in *in vitro* co-culture systems has facilitated the ability of the engineered DCs to induce T-cell activation and proliferation, giving insights into the immunogenicity of antigens that were used and the nature of presentation to the T-cells [35, 44, 58]. A coculture of MVA-infected dendritic cells within a mixed lymphocyte culture demonstrated cellular activation and an increase in IFN-*γ* production in both CD4^+^ and CD8^+^ T-lymphocytes. The ability of dendritic cells to induce proliferation and differentiation of Th1 cells has been observed [44]. Assessment of an effector versus memory T-lymphocyte response as a result of HIV-1 RNA introduction to dendritic cells has also been made possible. Autologous Gag-transfected dendritic cells were found to induce an increase in central memory CD8^+^ T-cells in a coculture with patient T-lymphocytes, as shown by detection of markers associated with various T-lymphocyte populations following coculture for 12 days [35]. Coculture experiments thus enable early assessment of T-lymphocyte differentiation under a variety of conditions in response to DCs manipulated ex vivo.

## 3. Variables and Therapeutic Outcomes of Clinical Trials

Many HIV-1 dendritic cell vaccines that have shown promise *in vitro* have reached clinical trials in which efficacy was investigated in patients with well-suppressed HIV-1 infections. The efficacy of immunotherapy can be expected to be dependent on numerous factors, including host genetics, whether patients remain on ART or discontinue ART after treatment, the necessary frequency of administration, the site of inoculation, and the severity of their immunocompromised state before initiating immunotherapy. An additional challenge in clinical trials of DC immunotherapy has been the genetic diversity of the HIV-1 quasispecies in an infected individual that develops after the initial infection [59–61]. Even during ART suppression of replication, there is a reduced but measurable level of mutation in proviral DNA that contributes to the diversity of HIV-1 genotypes within each individual [62]. Ongoing genotypic variation not only makes immunotherapy more challenging by altering the targets of therapy but also alters the spectrum of HIV-1-associated pathogenesis. Studies have uncovered mutations in genes encoding HIV-1 proteins, such as Tat and Vpr, that can be considered determinants and/or markers for altered HIV-1 pathogenic outcomes [63–68]. Minimizing the emergence of HIV-1 quasispecies during the course of infection has been recognized as an important objective of an effective dendritic cell immunotherapy. In addition, clinical trials vary in assays and endpoints used to measure clinical outcomes.

### 3.1. Patient Selection Criteria

In all clinical trials, the outcome of the trial can be influenced by the inclusion and exclusion criteria that govern patient recruitment into the trial. In the last ten years, even clinical trials which shared the common vaccine formulation design of DCs electroporated with HIV-1 mRNA have varied greatly in experimental setup and reporting of patient information (Table 1). One of the more important parameters for screening eligible patients for DC immunotherapy trials appears to be HIV-1 plasma viral load (generally <50 copies/mL) at the beginning of trial before vaccine administration. There has, however, been some variation on what has been considered “low” viremia upon entry into the trial; some studies instead reference residual viremia as being between 1 and 10 copies/mL [69–72]. This is further complicated by the need for maintaining stable suppression of viral load in recruited patients on ART alone. The likelihood of treatment failure (i.e., an inability to reduce viral load below the 50 copies/mL threshold) increases in patients with increased baseline viral loads (>150,000 copies/mL) at ART initiation [16]. In any case, the level of viremia at the start of DC immunotherapy may have an impact on the effectiveness of viral suppression subsequent to DC vaccination. Moreover, an association between the copy number and risk of viral rebound after treatment has been reported [73–75]. The absence of a consensus among different studies regarding the starting viremia in patients selected for trials likely complicates any comparative conclusions about viral rebound after DC vaccination in these trials.

Another variable in patient selection for clinical trials of DC vaccination is the patient's ability to control HIV-1 infection in the absence of ART. Many DC trials investigated efficacy in well-suppressed patients on ART. Without suppressive ART, however, 70-80% of HIV-1-infected patients progress to the Acquired Immune Deficiency Syndrome (AIDS) after a period of clinical latency [76, 77]. The rate of progression in these patients can vary and can sometimes be rapid. There are, however, smaller numbers of HIV-1-infected individuals known as long-term nonprogressors, or elite controllers, who are able to control infection in the absence of ART [78, 79]. It has been unclear how much the therapeutic outcome would differ between a long-term nonprogressor who has the ability to maintain normal CD4^+^ counts and low viremia without ART and individuals who are rapid progressors and developed AIDS much faster in the absence of ART. The remaining HIV-1-infected individuals falling outside this group included rapid progressors or elite controllers [76, 78–80].

Although the outcomes which considered measures of efficacy have differed in recent studies, induction of specific cytokines and antigen-specific T-lymphocyte responses were common endpoints. The ELISPOT (enzyme-linked immunosorbent spot) was used to indirectly assess the HIV-1 antigen-specific CD8^+^ T-lymphocyte response through the detection of granzyme B and IFN-*γ* release [38, 81, 82]. Additional outcomes included measurements of T-cell proliferation, changes in CD4^+^ T-lymphocyte count, and changes in peripheral blood viral load postvaccination, the latter to specifically check for viral reemergence.

### 3.2. Conduct and Outcomes of Recent Clinical Trials

A number of clinical trials have been conducted to evaluate the safety and efficacy of DC vaccination strategies. Outcomes of these trials suggested the potential and revealed the limitations of DC-based vaccinations against HIV-1. In addition, these trials highlighted important variables that must be considered in these types of clinical trials, including the impact of genotypic HIV-1 variation (viral quasispecies) on the vaccine effectiveness and the role of ART in determining postvaccination outcomes.

The AGS-004 investigational vaccine reached phase II clinical trials. In this vaccine, RNA encoding consensus sequences from Gag, Nef, Rev, and Vpr were electroporated into patient-derived dendritic cells along with CD40L mRNA [56]. Patients selected for the study were adherent to an ART regimen for at least three months prior to the beginning of the trial. They remained on ART and were administered the AGS-004 vaccine intradermally every four weeks for a total of four treatments. The results specifically showed an increase in CD8^+^ T-lymphocyte proliferative responses in seven of the nine subjects examined. This result was expected, as this vaccine approach was aimed at inducing an effective CTL response to eliminate infection.

A follow-up phase IIB study by Jacobson and colleagues was also completed with the AGS-004 vaccine candidate [36]. In this study, ART discontinuation in all subjects occurred 16 weeks after vaccine administration. The viral kinetics were more closely examined in this study. Unexpectedly, the kinetics appeared to be the same between the subject and placebo groups, suggesting that treatment did not prominently promote an antiviral effect oriented toward limiting viral replication or production. Similar to what was observed in the previous clinical study, however, induction of an HIV-1-specific effector memory CD8^+^ T-lymphocyte response occurred in patients who remained on ART. This response was further enhanced by additional treatments with the DC vaccine and was independent of the reemergence of virus with the discontinuation of ART [36].

The influence of variations in quasispecies between individuals on these suboptimal responses was addressed in a later modified study, AGS-004-003, in which patient selection criteria were limited to individuals who initiated ART suppression during acute HIV-1 infection (AHI) [83]. Patients in this study were administered the AGS-004 vaccine monthly, and generation of multifunctional effector CD8^+^ T-cell subtypes was analyzed after 3-4 doses. In these patients, expansion of multifunctional CD28-/CCR7-/CD45RA- effector CTLs producing TNF-*α*, IL-12, and IFN-*γ* occurred, at which point the patients underwent analytical treatment interruption. Although vaccine administration did not prevent long-term viral rebound, the expansion of these multifunctional CTLs strongly correlated with a longer time before viral rebound [83]. Additionally, patients in which time to viral rebound was the longest had differentiated effector CTL as opposed to central/memory CTLs, further suggesting that promotion of this transition in the CTL population may serve as an additional measure of efficacy. The results from this most recent trial with AGS-004 vaccination did not support the hypothesis that AGS-004-induced HIV-1 control would be more likely in patients suppressed during AHI but nonetheless verified the ability of ex vivo manipulated DCs to promote more robust CTL responses [83].

A similar trial with a Tat-Rev-Nef-Gag mRNA combination electroporated into DCs was performed on six individuals [56]. This study involved a subcutaneous injection of the vaccine along with the intradermal injections every four weeks and continued ART administration postvaccination. In this study, a vaccine-dependent induction of CD8^+^ T-lymphocyte cell effector responses was noted rather than a memory T-cell response. An increase in IFN-*γ* was observed only in response to the Gag antigen [56]. In light of these and other studies, the value of ART continuation or interruption postvaccination is not clear.

Interestingly, different results were produced by a slightly different study that involved ART discontinuation and use of HIV-1 lipopeptides covering epitopes from Gag, Nef, and Pol instead of antigens encoded by RNA. Both a CD4^+^ and CD8^+^ T-lymphocyte expansion was observed in all study participants, as well as prominent IL-2 and IFN-*γ* production by the CD4^+^ T-lymphocyte-specific response [59]. The contribution of CD4^+^ T-cells to the maintenance of an effective CTL response may thus have been overlooked in many DC vaccine clinical trials. Differences in outcomes between this trial and others may be attributed to differences in the forms of the antigens (particularly Nef- or Pol-derived peptides) as they may be more immunogenic [57]. Additionally, this study documented more detailed information about the patient population and noted that none of the subjects in the study had protective HLA (either B27 or B57) haplotypes, as these may contribute to efficiency of the antigen presentation underlying the desired immune response that was mounted. Furthermore, the study highlighted the need for continued ART, since the subjects that had ART discontinued during the vaccine treatment had a viral load much higher than the treated group that remained on ART [57]. As with the follow-up study on the AGS-004 vaccine, the reasons for the lack of success in controlling viremia in this clinical trial have just begun to be explored. A later study further characterized the importance of the CD4^+^ T-lymphocyte function in immunized patients following ART discontinuation and found a strong correlation between IL-2- and IL-13-producing CD4^+^ T-lymphocytes with lower viral loads in patients following ART discontinuation [84]. A further understanding of CD4^+^ T-cell function after immunization, in addition to CTL function, may help identify additional means for overcoming the inability to affect sustained control of viremia.

Among these studies, a dendritic cell vaccine was generally considered safe, with the most severe effects reported being flu-like symptoms and reactions at the injection site. The concern for autoimmunity was also not observed [38]. In fact, dendritic cell vaccines have been investigated for the treatment of autoimmune diseases [57, 85–88]. In the trials with HIV-1-infected individuals, the slight differences in patient criteria, such as the starting CD4^+^ T-cell count and site of injection, may have contributed to the variation in efficacy and may provide insights into the interdependent nature of the immune cells participating in host immune system response. This also highlights the need for other methods of ensuring proper delivery, which would inform the optimal choice of the route of injection. For example, Routy and colleagues [38] argued that intradermal administration would induce better migration through the lymph node relative to subcutaneous delivery. However, the study designs will likely be optimized through the performance of additional clinical trials. While viremia was not shown to be inhibited nor reduced in most trials, the enhancement of the CTL response may greatly help in preventing viral reemergence when combined with other drugs that specifically inhibit HIV-1 replication. The results of these recent studies ultimately highlight the challenge of eradicating HIV-1 using only one approach but suggest that DC vaccination may be a valuable part of a regimen that includes multiple therapeutic approaches.

## 4. Considerations for Optimizing DC Vaccines in Future Studies

The success rate of recent HIV-1 dendritic cell immunotherapy trials has been estimated to be no more than 38% as determined through a meta-analysis study [89]. Sustaining the induction of HIV-1-specific T-lymphocyte responses elicited by dendritic cell vaccines can be achieved through improvement not only of the design of autologous dendritic cells carrying HIV-1 antigens but also through understanding ways to avoid interfering signaling cascades initiated by other immune cells after delivery to the patient. For this reason, studies have examined the contributions of immunosuppressive T_reg_ cells with respect to reducing efficacy. These regulatory T-lymphocytes have primarily been of concern because they can either be naturally occurring or develop from naïve T-cells after interaction with dendritic cells and antigen [90–93]. Additionally, patient-specific variables, such as host genetics, need to be considered in determining the mechanisms driving the different results observed in these trials [94]. In a trial with inactivated HIV-1 loaded as a DC-based vaccine, a genetic screening of the subjects revealed polymorphisms in particular genes associated with the immune response (such as the MBL2 gene that is involved in immune recognition) in the subjects experiencing a weak response to the DC vaccine [54, 95]. In another study, a polymorphism in the CCR4-NOT transcription complex, subunit 1 (CNOT1) gene, was associated with an ineffective response, which may possibly be due to an indirect influence on the expression of genes involved in the inflammatory process [54, 94–96]. Such differences between patients are worthy of further investigation in order to obtain a more accurate prediction of vaccine efficacy in a patient group.

Potential strategies to enhance DC vaccine design include the incorporation of adjuvants as well as specific T-cell immunogens and other molecules that promote the necessary surface ligand interactions to overcome inefficient antigen presentation to CD4^+^ T-lymphocytes. A previous strategy for circumventing anticipated challenges in obtaining the desired immunogenicity was the use of the influenza A matrix protein as an adjuvant [19, 97]. Additional adjuvants, including adenosine deaminase (ADA), have contributed to both CD4^+^ and CD8^+^ T-cell proliferations after costimulatory molecule expression in dendritic cells [98, 99]. Similarly, an adjuvant combination of CD40L, CD70, and the constitutively active TLR4 (caTLR4) receptor (TRIMIX) was electroporated with HIV-1 antigens into dendritic cells in another study and was shown to cause enhanced IFN-*γ* secretion and an increase in HIV-1-specific CTLs after intranodal immunization in mice [100–103].

As many of the most recent studies have focused on mRNA electroporation as opposed to vector carriers, it appears this method of antigen loading has been highly reliable for delivering antigen to DCs. However, other delivery methods not yet examined may provide improved outcomes. Dendrimers as carriers of antigen have been proposed as a delivery method that will facilitate a more controlled antigen release mechanism, resulting in a more efficient response against HIV-1-infected cells [104]. The structure of a dendrimer, which consists of highly branched molecules extending from a core containing cavities and an amine group-rich surface, permits these macromolecules to act as nanocarriers in two ways: through attachment of a drug of choice onto the surface or encapsulating a drug in the internal cavities. Dendrimers have the advantages of low manufacturing costs and scalable synthesis [105]. Alternatively, a polylactic acid colloidal (PLA) nanoparticle was used for the delivery of p24 antigen to myeloid DCs (MD-DCs), which resulted in induction of DC maturation and increases in IFN-*γ* and IL-2 release and migration capacity. The same study also demonstrated that the use of nanoparticles for antigen delivery resulted in an increase in the proliferation of HIV-1-specific CTLs [106].

DC vaccination efficacy may also be enhanced by the administration of immunomodulatory therapies. Treatment with heterodimeric IL-15 (hetIL-15) was used recently to decrease HIV-1 RNA in the plasma, possibly through the maintenance of natural killer (NK) cells. Specifically, hetIL-15 allows for CTL targeting of infected cells in immune privileged sites within the secondary lymphoid tissues [107]. Aside from a suboptimal efficacy, however, some effects of this treatment included the induction of anti-inflammatory processes with an increase in IL-10 production and increased expression of programmed death-1 (PD-1) [108]. Furthermore, PD-1 has been suggested to contribute to HIV-1 disease progression and latent infection through a variety of mechanisms, including the upregulation of the master transcription factor BATF that has been associated with T-cell dysfunction [109, 110]. Inhibition of this immune checkpoint marker may greatly avoid failure associated with T-cell dysfunction in immunotherapy trials.

As many cancers also exhibit efficient evasion of immune recognition, it is possible that more successes can be achieved by applying lessons learned from cancer research. For example, the PD-1 inhibitors, nivolumab and pembrolizumab, have been investigated in cancer research because of their ability to boost the immune response. Both inhibitors have also been tested in HIV-1-infected individuals with cancer [111–113]. Since PD-1 has been considered an immune checkpoint or coinhibitory molecule, its inhibition of T-lymphocytes has been shown to allow for tumor or infected cell eradication by preventing the T-lymphocyte exhaustion that occurs due to overexposure to antigen [114–116]. Alternatively, a mechanism suggested to be the cause of success during DC vaccine administration in cancers is immunogenic cell death (ICD). Display of damage-associated molecular patterns (DAMPs), such as calreticulin, induces engulfment when bound by the CD91 receptor on phagocytes such as dendritic cells [111, 116]. The induction of DAMP expression by various stimuli has been suggested to enhance DC vaccines directed against certain cancers, as it promotes DC maturation and subsequent activation of CTLs specific to tumor cells [114, 117–120]. This process, if it can be applied as part of a viral therapeutic strategy, may enhance the efficacy of HIV-1-specific DC therapeutic vaccines.

## 5. Maximizing HIV-1 DC Immunotherapy Using Combinatorial Approaches to Overcome Latency

Immunotherapy and control of HIV-1 reemergence postvaccination represent a lower degree of difficulty relative to the more challenging goal of achieving complete eradication of the virus in HIV-1-infected individuals [121]. The replication cycle of HIV-1 is a relatively challenging target for chemotherapeutic approaches that may be proposed as the basis of a functional cure within the near future. ART alone is impractical as a means of eradication, as it has been estimated that over 60 years of ART would be necessary to completely eradicate infected cells from the body [122, 123].

Perhaps, one of the central elements associated with achieving the goal of HIV-1 eradication is the issue of latent infection and the necessity of reactivating and/or eliminating the latently infected, persistent cell reservoir. While immunotherapy could be used to promote immune responses against productively infected cells, cells harboring latent HIV-1 proviral DNA are much more challenging targets since they express few or no viral proteins. In addition, factors that contribute to latency may be outside the scope of immunotherapeutic approaches. For example, variations in the HIV-1 long terminal repeat (LTR), which are associated with clinical disease severity and might also be linked to the maintenance of viral latency [124], would not be addressed by an ex vivo DC vaccination approach, since the LTR does not code for proteins that could be targeted by immunotherapy. As one approach to overcome these challenges, DC vaccines may need to be combined with latency reversing agents (LRAs) to allow for more effective purging of the viral reservoirs. Interestingly, the inability of many DC vaccine trials to achieve a reduced HIV-1 RNA load after vaccination may be due to either an increase in virus production caused by the vaccine or killing of infected cells by the CTLs [23].

**Figure 3:**
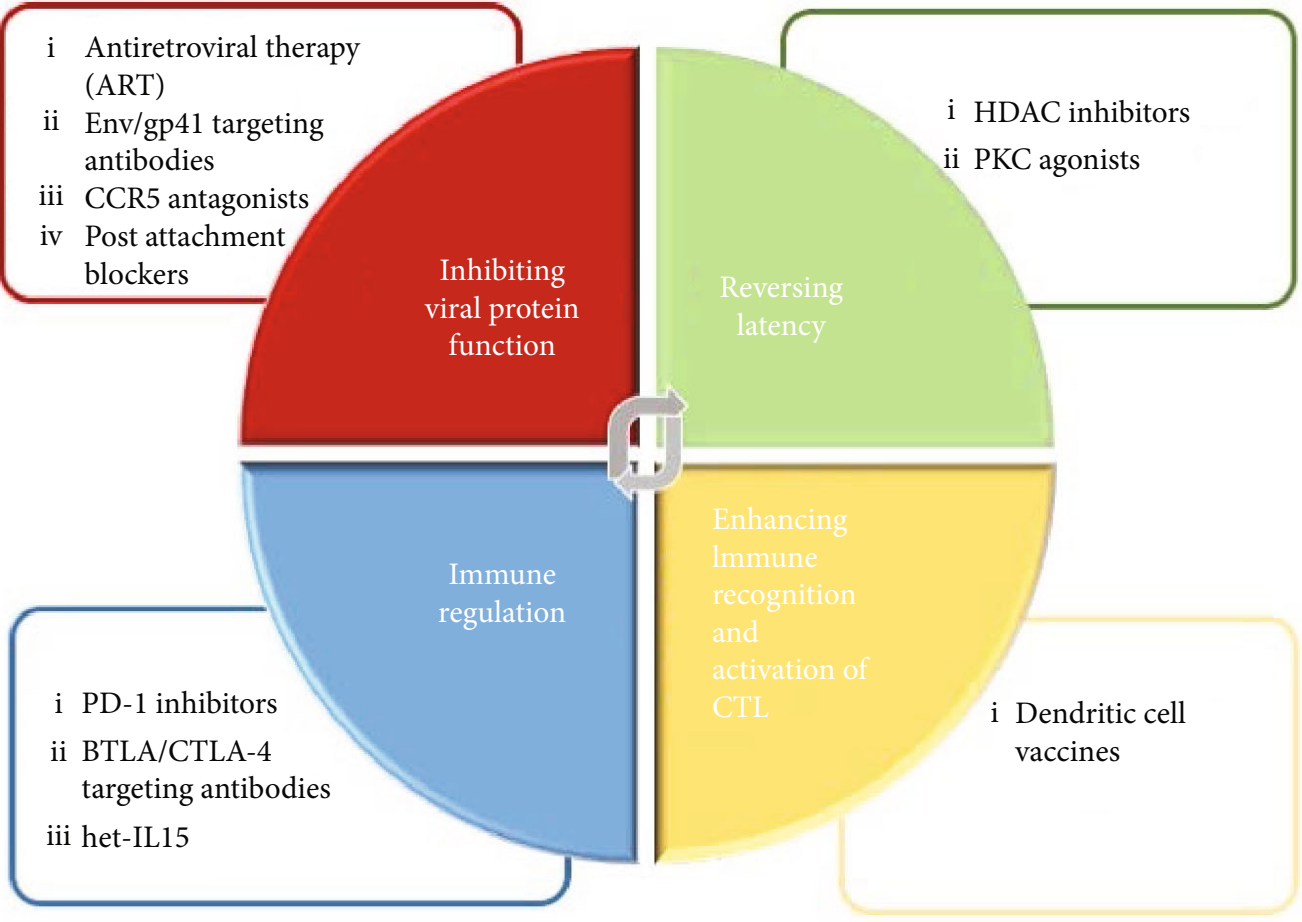
Summary of strategies currently being investigated for HIV-1 treatment. The design of a long-term HIV-1 treatment is generally focused on four approaches; reversing latency, inhibiting T-cell exhaustion markers, inhibiting viral protein function, and enhancing HIV-1 antigen recognition and immune regulation. Dendritic cell immunotherapy in the form of DC vaccines allows for a more unique approach to treating HIV-1 infection by specifically inducing better recognition by and activation of CTLs. Other therapies for HIV-1 infection can be combined to compensate for shortcomings of a single treatment option to provide optimal control of HIV-1 disease progression, viral resistance, and the spread of infection.

Immunomodulatory drugs such as thalidomide and pomalidomide can potentially increase the chances of reversing latency when utilized with dendritic cell vaccines. These drugs were found to enhance the lytic activity of HIV-1-specific CTLs as well as acting on the humoral branch of the immune system to reactivate Epstein–Barr Virus (EBV) in resting memory B cells [125, 126]. Latency reversal reagents such as the histone deacetylase inhibitor (HDACi) vorinostat can rapidly cause a change in gene expression that results in the induction of HIV-1 gene transcription in the resting CD4^+^ memory T-lymphocyte reservoir [127–130]. Additionally, the HIV-1 accessory protein Tat has been found to contribute greatly to the reactivation of latent infection. When Tat is present, the HDAC inhibitors vorinostat and panobinostat as well as the bromodomain inhibitor JQ1 can further increase HIV-1 gene transcription [131, 132]. While this was demonstrated for these drugs when used individually, it is important to note that multiple LRAs may need to be used in combination as they are unlikely to be efficient *in vivo* when used individually [133]. However, it remains to be determined if LRAs would still need to be used in combination with a dendritic cell vaccine against HIV-1. In conclusion, there appear to be multiple avenues for combination strategies designed to either enhance control of HIV-1 infection or lead to a cure for HIV-1 infection through complete eradication of the virus (Figure 3).

## 6. Conclusions

The dendritic cell immunotherapy approach may still be a promising method of immunotherapy targeted at increasing the efficacy of an individual's own immune response against HIV-1 infection. By allowing for the manipulation of autologous antigen-presenting cells in various ways, this approach has the potential advantages of reversing the antigen presentation dysfunctions observed in HIV-1-infected individuals as well as achieving stimulation of both CD4^+^ and CD8^+^ T-cell responses. Because of these advantages, DC immunotherapy has the potential to be used for long-term control of HIV-1 infection or even complete eradication of HIV-1 from infected individuals. One of the primary challenges in eradicating HIV-1 infection appears to be establishing long-term control of viral replication while simultaneously eliminating the viral reservoirs and preventing viral reemergence due to escape mutations. The multitude of effects elicited, unlike other therapies that rely on a specific mechanism of HIV-1 inhibition (such as entry inhibition), provides DC vaccines with the potential to provide a more long-term therapeutic effect, even if that effect is ART-free control of HIV-1 infection (a “functional cure”) rather than eradication. Additionally, their safety profiles allow them to be readily used as possible synergistic or supplementary therapies with other approaches to provide maximal protection against HIV-1 disease progression or virus eradication.

However, the varying levels of effectiveness reported among the different DC vaccination trials over the last ten years suggest a need to standardize measures of efficacy in order to better understand the potential of the DC vaccine to induce long-term immune protection and viral control in the absence of ART. Accordingly, standardization of patient selection criteria along with consistent and thorough reporting of patient background would simultaneously determine possible host influences on achieving long-term viral suppression. After additional trials focusing on the optimized administration of autologous cells carrying various forms and combinations of HIV-1 antigens, HIV-1-specific DC immunotherapy has the potential not only to offer ART-free control of HIV-1 infection but also to be a part of future eradication strategies that merit further investigation and development.

## Figures and Tables

**Table 1:** List of DC immunotherapy clinical trials within the last ten years that utilized dendritic cells electroporated with HIV-1 mRNA and their therapeutic outcomes. Clinical trials differed by antigen loaded in dendritic cells, inclusion of an adjuvant, method of administration to the subject, required adherence to ART, and measured changes in immune responses subsequent to vaccine administration.

	Patient selection criteria			Study design				Postvaccination efficacy measures		
Study	Starting CD4^+^ T count	Starting viral load	Patient medical history	Autologous antigen	Method of introduction	Dose frequency	Duration of study	Effect on CTL response	Associated cytokines produced	Clinical outcome conclusions
Routy et al. [38] AGS-004 Phase II	≥350 cells/mm^3^	<200 copies of RNA/mL	ART suppressed No coinfection with hepatitis B or C No use of systemic steroids or hydroxyurea	Gag, Nef, Rev, and Vpr and CD40L	Intradermal	Every 4 weeks in combination with ART	≥12 weeks	CD8^+^ T-cell proliferative responses were elevated at 4 and/or 8 weeks for all subjects	Not reported	Full or partial responses specific for the AGS-004 presented HIV antigens occurred in most subjects
Jacobson et al. [36] AGS-004 Phase IIB	>450 cells/mm^3^	<50 copies/mL	ART suppressed (≥3 months)	Gag, Nef, Rev, and Vpr and CD40L	Intradermal	Every 4 weeks ART interruption at week 16 for 12 weeks	32 weeks	Increase of CD28/CD45RA^−^CD8 effector memory T-cell response after 2 doses	IL-2, IFN-*γ*, TNF-*α*	No antiviral effect Increased CTL response did not correlate with reduced viral load
Van Gulck et al. [56] Phase I/II	>200 cells/mm^3^	<50 copies/mL	ART suppressed	Gag and a chimeric Tat-Rev-Nef	Half subcutaneous, half intradermal	Every 4 weeks in combination with ART	≥18 weeks	Increase of HIV-specific CD8^+^ T-cell responses	Gag-specific IFN-*γ* most significant	Improved antiviral response associated with Gag-specific IFN-*γ* response
Allard et al. [39] NTR2198 Phase I/IIa	>500 cells/mm^3^	≤50 copies/mL	ART suppressed No coinfection with hepatitis B or C No HIV-1 seroconversion within one year prior to study No history of lymph node irradiation	Tat, Rev, or Nef	Half subcutaneous, half intradermal	Every 4 weeks ART interruption at 14 weeks for ≤96 weeks	>96 weeks	Induction of Tat-, Rev-, and Nef-specific IFN-*γ* response	Gag-specific IFN-*γ* most significant	No correlation between any of the T-cell responses and the time remaining off cART was found No considerable decrease in plasma viral load
Gandhi et al. [50] PARC002 Pilot study	>300 cells/mm^3^	<50 copies/mL	ART suppressed No HCV antibody positivity No history of cardiac, gastrointestinal, hepatic, renal, pancreatic, neurologic, or autoimmune disease	Gag, Nef, and neoantigen KLH	Intradermal	At weeks 0, 2, 6, and 10	48 weeks	No Gag-specific or Nef-specific IFN-*γ* response	Overall levels of IL-2, IFN-*γ*, and TNF-*α* inconclusive	No Gag-specific or Nef-specific IFN-*γ* response Short-lived 2.5-fold increases in CD4^+^ T-cell proliferative response from baseline to HIV-1 Gag and 2.3-fold increase to HIV-1 Nef
